# The complete chloroplast genome sequence of *Acidosasa gigantea* (Bambusoideae: Arundinarieae): an ornamental bamboo species endemic to China

**DOI:** 10.1080/23802359.2020.1726224

**Published:** 2020-02-11

**Authors:** Xiao Zheng, Meng Yang, Yu-long Ding, Shu-Yan Lin

**Affiliations:** Co-Innovation Center for Sustainable Forestry in Southern China, Bamboo Research Institute, College of Biology and the Environment, Nanjing Forestry University, Nanjing, P. R. China

**Keywords:** Arundinarieae; *Acidosasa gigantea*, genome skimming, chloroplast genome, phylogenetic relationship

## Abstract

We, at first, fully characterized the complete chloroplast genome of the woody bamboo *Acidosasa gigantea* using genome skimming and focused on comparative analyses among *Acidosasa* and *Indosasa*. This newly sequenced chloroplast genome (GenBank NO. MN917206) is a typical circular structure with 139,711 bp in length and comprises of an 83,295 bp large single-copy (LSC) region, a 12,824 bp small single-copy (SSC) region, and a pair of 21,796 bp inverted repeats (IRs). The GC content of *A. gigantea* is 38.9% and the sequences contained 132 unique genes were successfully annotated, including 39 tRNA genes, 8 rRNA genes, and 85 protein-coding genes. Phylogenetic analysis shows that *A. gigantea* is highly clustered in the *Arundinaria* clade (clade VI) of Arundinarieae, a sister of the clade of *Acidosasa purpurea* and was not clustered on the same branch as *Indosasa sinica*. Therefore, it is more accurate to classify *A. gigantea* into *Acidosasa*.

Bamboo (subfamily Bambusoideae) is a large clade of Poaceae. There are more than 88 genera and 1400 species worldwide (Chen et al. 2006). However, the classification of some species is still controversial in traditional classification. *Acidosasa gigantea* is a potentially ornamental bamboo species for its lush foliage and straight stalks and can also be used as wood materials. The reconstruction of phylogenetic relationships among families, genera and even species based on the information provided by the whole chloroplast genome sequence has gradually become a new trend and many good results have been obtained (Ma et al. 2014; Nie et al. 2020). To date, there are limited species of plastid genomes that have been reported in bamboo plants, such as *Gelidocalamus tessellatus* (Ma et al. 2014), *G. xunwuensis* (Zhang et al. 2019), *Phyllostachys reticulate,* and *Ph. edulis* ‘Pachyloen’ (Huang et al. 2020).

*Acidosasa gigantea* studied in this paper was originally named *Sinobambusa gigantea* T. H. Wen in 1983 (Wen 1983) and then changed to *Indosasa gigantea* (T. H. Wen) T. H. Wen due to the discovery of 6 stamens in flowering in 1991 (Wen 1991). In addition, combined with the inflorescence type, Xie classified it in *Acidosasa*, which is *A*. *gigantea* (T.H. Wen) Q.Z. Xie & W.Y. Zhang (Xie and Chen 1993). Since then, there were disputes about problem of attribution of *A*. *gigantea* in the traditional classification. Here, we firstly obtained the complete chloroplast genome of *A*. *gigantea* by the method of genome-skimming sequencing. To some extent, that is a valuable resource for further studies in *Acidosasa* which could play a very important role in the unclear ownership and confused bamboo classification.

The young and healthy leaf samples of *A*. *gigantea* were collected from the bamboo garden of Jiangxi Agricultural University, China (28°45′40″N, 115°49′31″E). Voucher specimens (Hnj36052) were deposited at the herbarium of the College of Forestry, Jiangxi Agricultural University, China. Illumina paired-end (PE) library was prepared and sequenced in the Kunming Institute of Botany, Chinese Academy of Sciences (CAS) in Kunming, China. SPAdes 3.13.0 (Bankevich et al. 2012) and Geneious 9.0.5 (http://www.geneious.com/) were used to splice and assemble all contigs of the chloroplast genome sequence. Then, annotation of the assembled chloroplast genome was performed using the webserver DOGMA (Wyman et al. 2004) and detected simple sequence repeats (SSR) by MISA (http://pgrc.ipk-gatersleben.de/misa).

The complete chloroplast genome sequence together with gene annotations of *A*. *gigantea* were submitted to the GenBank under the accession number of MN917206. It is a typical circular structure with 139,711 bp in length and comprise of an 83,295 bp large single-copy (LSC) region, a 12,824 bp small single-copy (SSC) region, and a pair of 21,796 bp inverted repeats (IRs). Its GC content is 38.9% and the sequences contained 132 unique genes were successfully annotated, including 39 tRNA genes, 8 rRNA genes, and 85 protein-coding genes.

To determine the phylogenetic status of *A*. *gigantea*, the complete chloroplast genomes of 28 trib. Arundinarieae and 3 outgroup species were downloaded from NCBI (Figure 1). RA × ML 8.2.8 (Stamatakis 2014) and MrBayes 3.2.6 (Ronquist and Huelsenbeck 2003) were used to generate the maximum likelihood phylogenetic tree and the Bayes tree. The results showed that *A. gigantea* is highly clustered in the *Arundinaria* clade (VI) of Arundinarieae, a sister of the clade of *Acidosasa purpurea* and was not clustered on the same branch as *Indosasa sinica*. Therefore, this paper considers *A. gigantea* is more accurately classified into *Acidosasa* based on the analysis of the complete chloroplast genome of *A. gigantea*.

**Figure 1.**
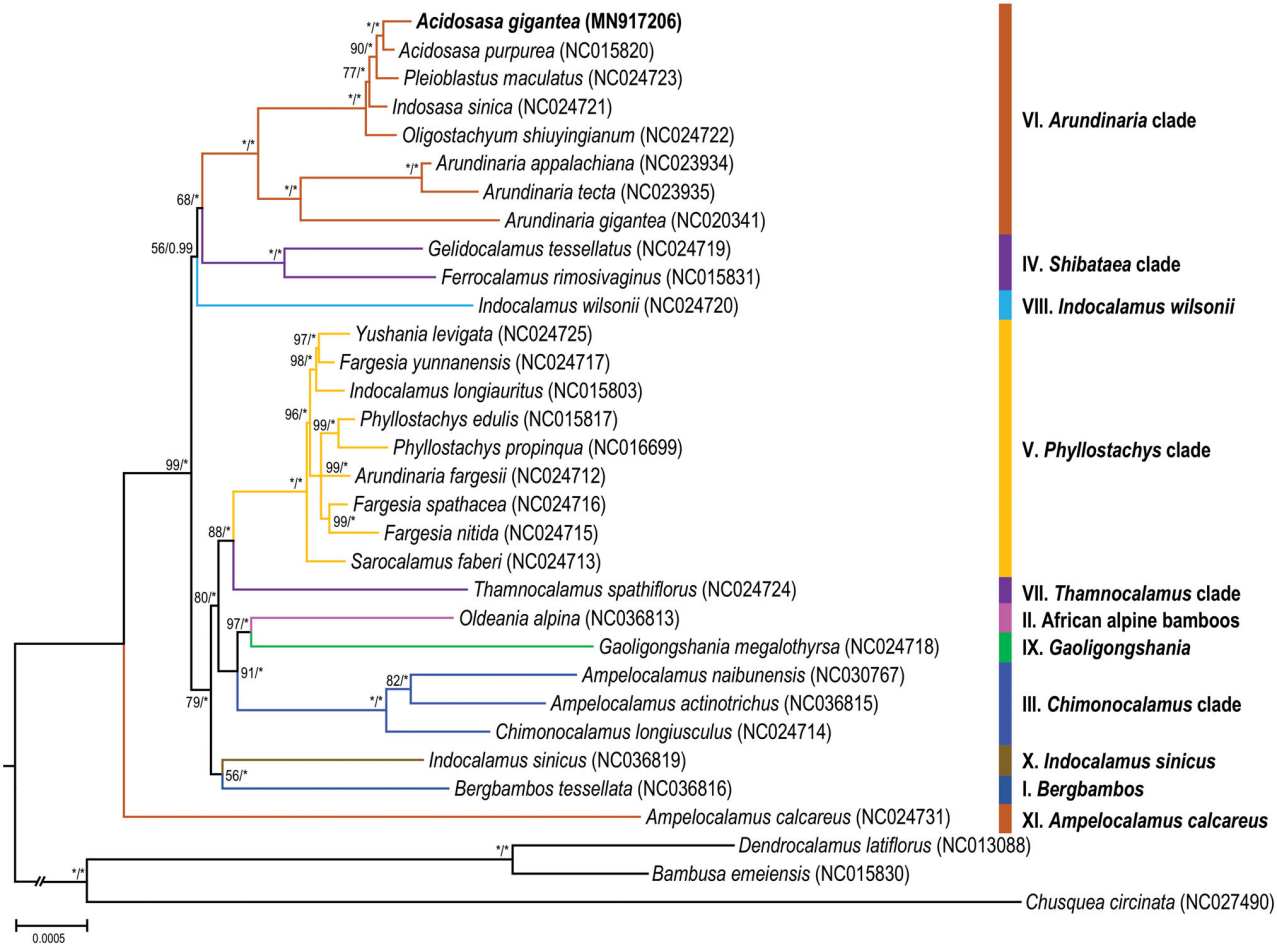
Maximum-likelihood phylogenetic tree based on complete chloroplast genomes from 31 bamboo species. Colored branches indicate the 11 Arundinarieae lineages (I–XI). Numbers above branches indicated the maximum likelihood bootstrap support and the Bayesian posterior probabilities, respectively. Asterisks indicate 100% bootstrap support or 1.0 posterior probability. Hyphens indicate the bootstrap support or posterior probability lower than 50% or 0.5.
